# Structures in Tetrahydrofolate Methylation in Desulfitobacterial Glycine Betaine Metabolism at Atomic Resolution

**DOI:** 10.1002/cbic.201900515

**Published:** 2019-11-18

**Authors:** Thomas Badmann, Michael Groll

**Affiliations:** ^1^ Center for Integrated Protein Science Munich (CIPSM) Department of Chemistry Technische Universität München Lichtenbergstrasse 4 85748 Garching Germany

**Keywords:** anaerobic bacteria, cobalamin, glycine betaine metabolism, methyl transfer, tetrahydrofolate

## Abstract

Enzymes orchestrating methylation between tetrahydrofolate (THF) and cobalamin (Cbl) are abundant among all domains of life. During energy production in *Desulfitobacterium hafniense*, MtgA catalyzes the methyl transfer from methylcobalamin (Cbl‐CH_3_) to THF in the catabolism of glycine betaine (GB). Despite its lack of sequence identity with known structures, we could show that MtgA forms a homodimeric complex of two TIM barrels. Atomic crystallographic insights into the interplay of MtgA with THF as well as analysis of a trapped reaction intermediate (THF‐CH_3_)^+^ reveal conformational rearrangements during the transfer reaction. Whereas residues for THF methylation are conserved, the binding mode for the THF glutamyl‐*p*‐aminobenzoate moiety (THF tail) is unique. Apart from snapshots of individual reaction steps of MtgA, structure‐based mutagenesis combined with enzymatic activity assays allowed a mechanistic description of the methyl transfer between Cbl‐CH_3_ and THF. Altogether, the THF‐tail‐binding motion observed in MtgA is unique compared to other THF methyltransferases and therefore contributes to the general understanding of THF‐mediated methyl transfer.

## Graphical abstract

**Enzyme catalysis in action**: In the glycine betaine metabolism of *D. hafniense*, MtgA catalyzes methyl transfer from methylcobalamin to tetrahydrofolate (THF). Using protein X‐ray crystallography combined with mutagenesis and enzymatic activity assays, we were able to obtain mechanistic insights into this regioselective methylation. Structural snapshots of MtgA during catalysis reveal discrepancies towards other THF methyltransferases.

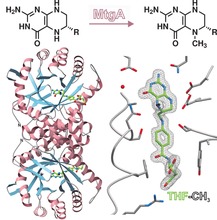

Enzymatic reactions involving the cofactor tetrahydrofolate (THF) are found ubiquitously throughout nature and support various transfer reactions of reduced single carbon species.^1^ THF methyl transfer is often dependent on cobalamin (Cbl), combining a simplistic reaction with two highly sophisticated cofactors.^2^ This raises the questions of how the transfer of the methyl group is carried out mechanistically and why such cofactor complexity is required.

The most prominent example of methyl transfer from THF to Cbl takes place in methionine biosynthesis, where the CH_3_ group from *N* ^*5*^‐methyltetrahydrofolate (THF‐CH_3_) is used to convert homocysteine into methionine via methylcob(III)alamin (Cbl‐CH_3_).^3^ Yet, THF‐ and Cbl‐coupled methyl transfer has been shown to be widely distributed. In the Wood–Ljungdahl pathway, acetogenic anaerobic microbes can grow on CO_2_ and methylated substrates by producing acetyl‐CoA by THF‐ and Cbl‐coupled methyl transfer.^4^ In addition, variations of these Cbl‐dependent enzyme systems exist that accommodate different methyl‐group sources and acceptor molecules for specific metabolic challenges.^5^ For instance, the O‐demethylation of phenyl methyl ethers as well as N‐demethylation of the quaternary amine glycine betaine (GB) are used for energy production as well as carbon assimilation in acetogenic bacteria.^6^ *Desulfitobacterium hafniense*, a non‐acetogenic organism, has been found to employ these precursors solely for energy production through anaerobic respiration.^7^ In the case of the GB pathway, this organism uses a series of enzymes, MtgB, MtgC, and MtgA, to transfer one methyl group via Cbl to THF (Scheme 1).^8^ MtgA catalyzes methyl transfer from Cbl‐CH_3_ to THF and lacks sequence identity towards solved protein structures.^9^, ^10^, ^11^ Herein, we present X‐ray structures of THF‐ and THF‐CH_3_‐bound MtgA that allow us atomic insights into the reaction trajectory of this catalyst. Site‐directed mutagenesis combined with activity assays identified a unique locking mode of the THF glutamyl‐*p*‐aminobenzoate moiety (THF tail).

**Scheme 1:**
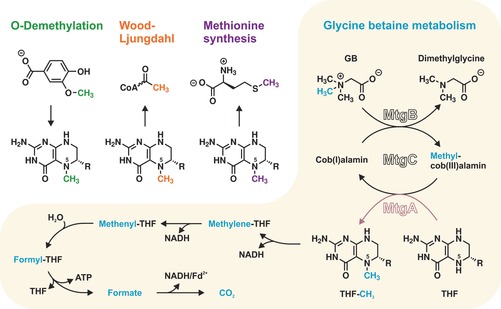
Top left: Physiological pathways involving methyl transfer between Cbl and THF. Yellow box: In microbial glycine betaine (GB) metabolism, the methyl group (blue) is abstracted from GB and transferred to the *N* ^*5*^ atom of THF. In *D. hafniense*, THF‐CH_3_ is formed by MtgA (pink) and further catabolized for energy production.^6^, ^8^ The glutamyl‐*p*‐aminobenzoate moiety of THF is abbreviated as R.

MtgA was cloned and heterologously expressed in *Escherichia coli* K12. The enzyme was purified to homogeneity and co‐crystallized with THF and THF‐CH_3_ (Figure S1, Tables S1 and S2 in the Supporting Information). Structure elucidation was performed by experimental phasing with selenomethionine‐labeled protein. THF and THF‐CH_3_‐bound complexes were solved at a resolutions of 1.35 Å (PDB ID: 6SJ8) and 1.55 Å (PDB ID: 6SK4), respectively (Table S3). MtgA assembles as a homodimer with a contact area of 1870 Å^2^;^12^ this agrees with retention times from size‐exclusion chromatography. The topology of each subunit is characterized by a funnel of eight parallel β‐sheets connected by α‐helices on the outside that form a complex of two perpendicularly oriented TIM barrels (Figure 1 A).^13^ The structure contains two additional small antiparallel β‐sheets at the N terminus. Compared to the typical (β/α)_8_ architecture, β‐sheet 7 in MtgA is replaced by a random coil and two small α‐helices ranging from residue 218 to 238 (Figure S2). The active‐site cavity is located inside the hydrophilic core of each barrel (Figure 1 B).

**Figure 1:**
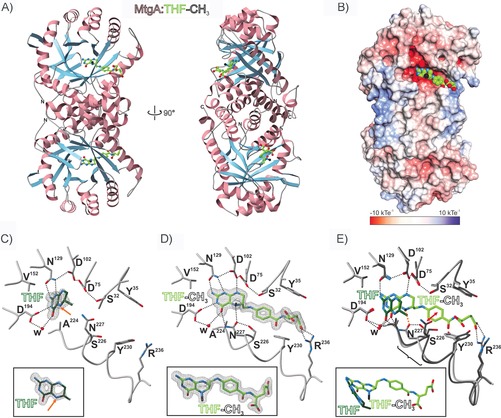
Crystal structures of MtgA in complex with THF and THF‐CH_3_. A) Top and side views of MtgA bound to THF‐CH_3_. The homodimer adopts a TIM barrel fold (helices: pink, β‐strands: blue, coils: grey). B) Surface charge distribution of MtgA. Cosubstrate coordination takes place in a solvent‐exposed binding cavity. C) MtgA in complex with its substrate THF. Residues engaged in cofactor binding are depicted as grey sticks and labeled by one‐letter code. The 2 *F*_o_−*F*_c_ electron density map (grey meshes, contoured to 1.0 σ) is shown for the ligand in two orientations (icon below). Only defined, inflexible moieties are depicted in the THF stick model. H‐bonds are drawn as black dotted lines. The orange arrow points to the *N* ^*5*^ atom where methylation takes place. D) MtgA bound to THF‐CH_3_ highlights a trapped reaction intermediate state (THF‐CH_3_ is sp^3^ hybridized). E) Structural superposition of MtgA:THF (dimmed) with MtgA:THF‐CH_3_ (bright) reveals a 35° tilt of the ligand. THF‐CH_3_ binding shifts Asn227 of MtgA by 2 Å (black bracket) to circumvent a clash with the THF tail (red double arrow). See also Figures S2–S5 for further structural illustrations of MtgA.

Intriguingly, the X‐ray structure of the MtgA:THF complex illustrates electron density only for the pterin moiety of the ligand; the THF tail seems to be flexible (Figure 1 C). The identified binding site of MtgA consists of the residues Ser32, Asp75, Asp102, Asn129 and Asp194, which stabilize the aromatic ring system of THF by hydrogen bonds. The close proximity of the acidic residues Asp75 and Asp102 towards each other is most striking. Hereby, the strong interaction of Asp102 with the cofactor increases the negative partial charge at *N* ^*5*^ of THF and activates it for attack of the incoming Cbl‐bound CH_3_ group. These first mechanistic insights were confirmed by replacing Asp102 with alanine. In the MtgA D102A mutant, methyl transfer from Cbl‐CH_3_ to THF is abolished as compared to the wild‐type (WT) enzyme (Figure 2 B).

**Figure 2:**
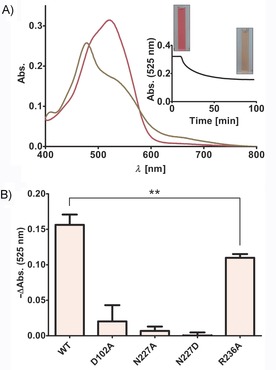
Enzymatic activity assays of WT and mutant MtgA. A) Spectra at 525 nm depict the conversion of Cbl‐CH_3_ to Cbl, which accompanies the methyl transfer catalyzed by MtgA. B) Comparison of catalysis (mean±SD; *n*=3) between WT and mutant MtgA. The reduced activity of MtgA R236A was verified by conducting an independent‐samples t‐test; *t*(4)=5.217, *p*=0.0064.

In contrast to substrate‐bound MtgA, the entire cofactor is defined in the MtgA:THF‐CH_3_ complex depicting extensive coordination within the amino acid environment in a rigid manner (Figure 1 D). The overall binding motif of the pterin ring remains similar to the substrate‐bound structure (Figure 1 C). However, the THF benzoate moiety is now involved in a π‐stacking network including Tyr230, Phe243, Phe251, and Trp272 on one side, as well as Tyr35 and His38 on the other. While the functional groups of the THF tail engage water‐coupled hydrogen bonding interactions with adjacent residues, the terminal γ‐carboxylate forms salt bridges with Arg236 that coordinate the cofactor in an extended conformation (Figure 1 D). Mutation of Arg236 to alanine resulted in impaired enzymatic activity (Figure 2 B), thus implying that fixation of the THF tail during methylation is involved in catalysis. Besides this fixation, the superposition of both ligand structures illustrates a rotation of the pterin moiety during the reaction of approximately 35°. In both poses, the aromatic ring system is well stabilized and most active‐site protein residues remain in their original position (Figure 1 E). In contrast, the benzoate group of THF‐CH_3_ displaces the carboxamide side chain of Asn227 by 2 Å (Figure 1 E, red double arrow). This conformation shift causes helix α7′ to collapse (Figure S2), which is compensated for by the formation of additional hydrogen bonds between *N* ^*5*^, Asn227, and Ser226 (Figure 1 E, yellow dotted line). Thus, the replacement of the typical TIM barrel β‐sheet 7 with helix α7′ (Figure S2) appears to be a necessary adjustment for efficient catalysis in MtgA.

In the MtgA:THF‐CH_3_ complex, the cofactor exhibits tetrahedral sp^3^ hybridization at its methylated amine *N* ^*5*^. As planar sp^2^ hybridization would be expected in this position of the final product THF‐CH_3_, the X‐ray structure must depict a trapped protonated reaction intermediate (Figure 1 D, E). As a result of its performed swinging motion, Asn227 is in position to form a strong hydrogen bond with the *N* ^5^ atom of (THF‐CH_3_)^+^. Notably, the active site lacks residues to deprotonate the (THF‐CH_3_)^+^ intermediate; this decreases the conjugated electron system within the pterin ring system. Supported by the inactive N227A and N227D mutants, we propose that proper stabilization of the tetrahedral *N* ^*5*^ state of (THF‐CH_3_)^+^ is fundamental for catalyzing the methyl transfer. (Figure 2 B).

X‐ray structure analysis of mutant MtgA in complex with THF‐CH_3_ gave further insights into the reaction trajectory. Whereas the N227A:THF‐CH_3_ complex (1.8 Å, PDB ID: 6SJS) features a water molecule in place of the carboxamide side chain as well as planar sp^2^ hybridization at *N* ^*5*^ (Figure S4 B), the D102A:THF‐CH_3_ structure (1.95 Å, PDB ID: 6SJO) displays undefined electron density around *N* ^*5*^, thus indicating fluctuation between sp^2^ and activated sp^3^ hybridization (Figure S4 A). Therefore, both mutants mimic product formation at a late reaction state. On the other hand, the WT MtgA:THF‐CH_3_ structure illustrates an intermediate that forms immediately after CH_3_ transfer from Cbl‐CH_3_ to THF (Figures 1 D and S4). Altogether, the presented crystallographic snapshots depict enzyme catalysis in action at atomic resolution.

Despite its individual sequence, MtgA shares the TIM barrel fold with a couple of Cbl‐dependent THF methyltransferases. These include methionine synthase (MetH) and the THF‐binding component from the carbon‐fixating Wood–Ljungdahl pathway in acetogenic bacteria (MeTr).^10^, ^11^, ^14^, ^15^ The desulfitobacterial methyl group acceptor protein MT2DH, which plays a role in the catabolism of aromatic methyl ethers (Scheme 1), shows the highest structural similarity with MtgA (r.m.s.d.: 2.9 Å, sequence identity: 13 %, *Z* score: 20.2; Figure S3).^9^, ^16^ In agreement with MT2DH and contrast to MetH and MeTr, MtgA catalyzes methyl transfer from Cbl‐CH_3_ to THF (Scheme 1 A). Regardless of the direction of methyl transfer, residues involved in catalysis and binding of the pterin ring are conserved. Moreover, a protonated THF intermediate seems to be a common feature of Cbl‐dependent THF methyltransferases.^17^ Nonetheless, MtgA has unique features. Whereas MT2DH lacks conformational rearrangements between THF and THF‐CH_3_‐bound states,^9^ the catalytic Asn of the more distantly related MeTr acts as gatekeeper for Cbl prior to entry of THF‐CH_3_.^15^ In MtgA, this Asn is involved in a swinging motion that is key to recognizing the proper folate cofactor and catalyzing the methyl transfer to its *N* ^*5*^ atom. Even though these enzymes share mechanistic principles, structural peculiarities are essential for efficient catalysis either in the catabolism of GB or other Cbl‐dependent THF methyl‐transfer pathways. Taken together, structural and biological characterization of MtgA supports the general understanding of biological systems employing the cofactor THF as a tool for methylation reactions.

## Conflict of interest

*The authors declare no conflict of interest*.

## Supporting information

As a service to our authors and readers, this journal provides supporting information supplied by the authors. Such materials are peer reviewed and may be re‐organized for online delivery, but are not copy‐edited or typeset. Technical support issues arising from supporting information (other than missing files) should be addressed to the authors.

Supplementary
