# Supplementary material for: Structures in Tetrahydrofolate Methylation in Desulfitobacterial Glycine Betaine Metabolism at Atomic Resolution
Source: Chembiochem. 2019 Nov 18;21(6):776–9. doi: 10.1002/cbic.201900515 (PMC7154762; doi:10.1002/cbic.201900515)
Supplement: Supplementary file 1 — Supplementary [file CBIC-21-776-s001.pdf]

CHEMBIOCHEM

Supporting Information

Structures in Tetrahydrofolate Methylation in Desulfitobacterial Glycine Betaine Metabolism at Atomic Resolution

Thomas Badmann and Michael Groll*[a]

cbic_201900515_sm_miscellaneous_information.pdf

Supporting information

1 Supporting method section 2
1.1 Cloning 2
1.2 Recombinant protein expression 2
1.3 Protein purification 2
1.4 Selenomethionine labeling of MtgA 3
1.5 Protein crystallization 3
1.6 Structure determination 4
1.7 Photometric activity assay 5
2 Supporting figures 6
3 Supporting tables 9
4 Supporting references 11

1 Supporting method section

1.1 Cloning

The gene sequence of MtgA from *D. hafniense* (Uniprot: Q24SP6; amino acid residues 2 -306) was adapted for recombinant expression in *E. coli* and obtained from Eurofins Genomics (Ebersberg, Germany). MtgA was amplified via PCR and cloned into a modified pET-28b(+) vector (Invitrogen, Darmstadt, Germany) containing an N-terminal His_6-SUMO protein construct [Smt3p from *Saccharomyces cerevisiae*] using the restriction sites BamHI and PstI (pET28bSUMO-Dh_MtgA). Point mutations were introduced using the QuikChange II site-directed mutagenesis kit (Agilent Technologies, Waldbronn, Germany) according to manual instructions. Cloned sequences were confirmed by Sanger sequencing (Eurofins Genomics, Ebersberg, Germany).

1.2 Recombinant protein expression

E. coli BL21 (DE3) cells (New England Biolabs, Ipswich, USA) were transformed with the derived constructs. A single colony was inoculated in 50 mL LB medium supplemented with 50 µg/mL kanamycin and grown at 37 °C over night. 3 L LB medium (containing 50 µg/mL kanamycin) were cultivated with the pre-culture in a Fernbach flask and incubated at 37 °C to an OD_{600} of 0.6. After shifting the culture to 20 °C, gene expression was induced with 1 mM isopropyl-β-D-thiogalactopyranoside (IPTG). Expression was performed over night at 20 °C. Cells were centrifuged at 7,200 x *g* for 30 min, washed in 0.9 % sodium chloride solution, and pelleted again at 5,000 x *g* for 15 min, and stored at -20 °C.

1.3 Protein purification

Recombinant MtgA proteins were purified from *E. coli* BL21(DE3) via immobilized metal ion affinity (IMAC) and size exclusion chromatography (SEC) according to standard protocols. Pellets of *E. coli* BL21(DE3) containing the protein of interest were thawed and resuspended in 50 - 100 mL buffer A (100 mM Tris/HCl (pH 7.5), 500 mM NaCl, 10 % (v/v) glycerol, 20 mM imidazole). 5 mg Pefabloc SC protease inhibitor (Roche, Basel, Swiss) were added and cells were disrupted by sonication on ice with a Branson digital Sonifier 250 (G. Heinemann, Schwäbisch Gmünd, Germany) (80 % amplitude, total time: 4 min, pulse on/off: 1 s). The lysate was centrifuged (40,000 x *g*, 30 min, 4 °C) and the supernatant applied onto a 5 mL HisTrap HP column (GE Healthcare, Freiburg, Germany) (flow rate 5 mL/min), pre-equilibrated in buffer A using an ÄKTA prime plus (GE Healthcare, Freiburg, Germany). The column was washed with 100 mL buffer A and subsequently developed with a linear gradient from buffer A to buffer B (= buffer A with 500 mM imidazole) over 50 mL to elute the His_6-fused protein. The collected sample was treated with His_6-SUMO protease in a 1:100 molar ratio, dialyzed at 4 °C over night against 5 L buffer C (20 mM Tris/HCl (pH

7.5), 100 mM NaCl, 10 % (v/v) glycerol), and applied onto the HisTrap column (equilibrated with buffer A). The cleaved protein without His_6-tag was collected in the flowthrough and concentrated to a volume of 2 mL using a 10 kDa NMWL Amicon Ultra Centrifugal Filter (Merck Millipore, Burlington, Massachusetts, USA). Subsequently, the sample was centrifuged (20,000 x *g*, 10 min, 4 °C) and loaded onto a 200 16/60 pg HiLoad Superdex column (GE Healthcare, Freiburg, Germany) (equilibrated in buffer C) on an ÄKTA purifier system (GE Healthcare, Freiburg, Germany). Protein containing fractions were pooled and concentrated to at least 30 mg/mL using an Amicon Ultra Centrifugal Filter. For prolonged storage, MtgA wild type and mutants were flash-frozen in liquid nitrogen and stored at -80 °C.

1.4 Selenomethionine labeling of MtgA

Selenomethionine (SeMet)-labeled protein was expressed in *E. coli* BL21(DE3) using a modified protocol by van Duyne *et al*., 1993.[1] 5 mL LB medium, supplemented with 50 µg/mL kanamycin, were inoculated from a cryostock of *E. coli* BL21(DE3), transformed with pET28bSUMO-Dh_MtgA, and incubated at 37 °C for 8 h. 300 mL M9 medium (supplemented with 50 µg/mL kanamycin, 0.4 % glucose, 2 mM $MgSO_4$, 1 mg/L riboflavin/niacinamide/pyridoxine/thiamine, 50 mg/L EDTA, 8 mg/L $FeCl_3$, 0.5 mg/L $ZnCl_2$, 0.1 mg/L $CuCl_2$/$CoCl_2$/H_3BO_3, 16 mg/L $MnCl_2$ and traces of Ni_2SO_4) were inoculated with 300 µL (1:100) pre-culture and cultivated at 37 °C over night. Out of this second pre-culture, 3 L supplemented M9 medium were transferred and incubated at 37 °C to an OD_{600} of 0.6. 0.3 g lysine, 0.3 g threonine, 0.3 g phenylalanine, 0.15 g leucine, 0.15 g isoleucine, 0.15 g valine, and 0.15 g selenomethionine were added. After 15 min at 37 °C, expression was induced with 1 mM IPTG. Cells were shaken at 37 °C for 4 h, harvested at 7,200 x *g* (30 min), washed in 0.9 % sodium chloride solution, pelleted at 5,000 x *g* (15 min), and stored at -20 °C. SeMet-labeled MtgA was purified analogous to wild type enzyme. Purification buffers additionally contained either 5 mM β-mercapto ethanol (HisTrap column), or 5 mM DTT (Superdex column).

1.5 Protein crystallization

Protein crystals were obtained via sitting drop vapor diffusion in Intelli 96-well plates. Purified protein was diluted to a concentration of 15 mg/ml in buffer C, optionally supplemented with 10 mM of either THF or THF-CH_3 (both Merck, Kenilworth, USA). Commercially available initial screens (Qiagen, Venlo, Netherlands) were used to search for promising crystallization parameters. The buffer reservoir of each well of Intelli 96-well plates was filled with 50 µL buffer solution with the pipetting robot Phoenix (Art Robbins Instruments, Sunnyvale, USA). Droplets for vapor diffusion comprising 1:1, 2:1, or 3:1 ratios (0.2 µl + 0.2 µl, 0.2 µl + 0.1 µl, and 0.3 µl + 0.1 µl) of protein and reservoir solution, respectively, were prepared with the pipetting robot Oryx4 (Douglas Instruments, Berkshire, GBR). The

sealed plates were incubated for several days at 20 °C. Crystals were identified by using a transmission microscope with 10x magnification. Promising conditions were used as templates for fine screening individual components to further improve crystal quality and size. These experiments were designed on Intelli 96-well plates with the pipetting robot MICROLAB STARlet (Hamilton, Reno, USA) by rastering pH, precipitant-, salt-, and/or cofactor concentration of the original condition. Crystallization conditions of protein crystals that allowed structure determination are annotated in Table S2. Next, 1 µL cryoprotectant, consisting of 60 % (v/v) glycerol mixed 1:1 with corresponding reservoir solution, was added to crystal containing droplets. Crystals were mounted onto a cryo loop and vitrified in liquid nitrogen.

1.6 Structure determination

Diffraction datasets of MtgA were collected up to 1.35 Å resolution using synchrotron radiation at the X06SA-beamline, SLS, Villigen, Switzerland (Table S3). Recorded reflections were processed with the program package XDS.[2] MtgA predominantly crystallized in the orthorhombic space group $P2_12_12_1$. Experimental phases were obtained by single anomalous dispersion (SAD) methods using the peak absorption wavelength of a selenium derivatized crystal (λ = 0.979 Å, f' = -6.7; f'' = 5.5). Using a dataset to 1.9 Å resolution, we could locate 10 heavy atom sites with SHELXD.[3] Subsequent SHARP-SAD-phasing[4] and solvent flattening with the program DM[5] resulted in an electron density map with phases to 2.8 Å resolution. The quality was sufficient to model secondary structure elements by polyalanine residues. The initial model was transferred to the native dataset with a resolution of 1.35 Å by applying rigid body- and positional-refinement using REFMAC5.[6] The resulting electron density map allowed unambiguous identification of the entire MtgA-sequence. The model was completed in iterative rounds with the three-dimensional graphic programs COOT.[7] Temperature factors were anisotropically refined with restraints between bonded atoms using translation / libration / screw motion-parameters, yielding crystallographic values of R_{cryst} = 0.113 and R_{free} = 0.157 (Table S3). The model was confirmed to have superb stereochemistry in the Ramachandran plot with 99.3% of residues in the most favoured and 0.7% of residues in the additionally allowed regions. The asymmetric unit cell contains two MtgA subunits with only the two N-terminal amino acid residues being structurally disordered. Next, synchrotron diffraction datasets of wild type and mutant MtgA crystal structures were recorded and refined using the high resolution MtgA coordinates as starting model. To our surprise, all derived datasets with THF-CH_3 lack defined electron density for the ligand in chain B (Fig. S5). We could prove that this observation is a crystallographic artefact because each subunit in the determined MtgA:THF-CH_3 structure (1.55 Å resolution, space group $P2_1$) is complexed with the THF-CH_3 ligand.

1.7 Photometric activity assay

Enzymatic activities were determined by using a modified version of established protocols for cobalamin-dependent methyltransferases.[8,9] Conversion of Co(III) to Co(I), which accompanies methyl transfer from methylcob(III)alamin to THF, was determined photometrically. To prevent immediate oxidation to cob(III)alamin, the assay was performed under anaerobic conditions.

After bubbling with N_2 for 10 min, 1 mL 100 mM Tris/HCl (pH 7.5) was transferred to a sealable cuvette (Hellma, Müllheim, Germany), which was held under N_2-atmosphere. Measurements were performed in an Ultrospec 7000 photometer (GE Healthcare, Freiburg, Germany). The solution was blanked after addition of 500 µM THF. For recorded spectra, 50 µM Cbl-CH_3 were submitted and absorptions from 400 nm to 800 nm measured within 2 h after adding 1 µM WT MtgA. For quantification of enzymatic activity, the absorption was measured at 525 nm each second for 10 min before and 2 h after addition of 1 µM WT or mutant MtgA. Data collection was recorded until apparent completion of the reaction. For comparison of the R236A mutant with wild type MtgA, an unpaired, two-tailed t-test was performed and evaluated with Graph-Pad Prism (Version 5.02, GraphPad Software, San Diego, USA).

2 Supporting figures

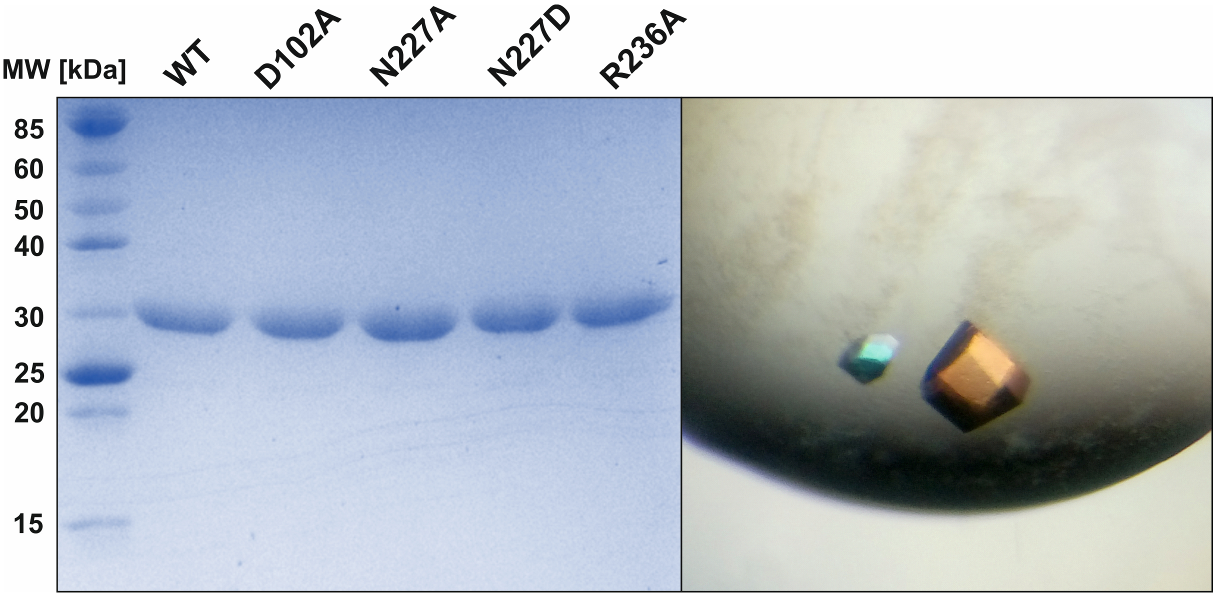

Figure S1. Protein purification and crystallization. Left) SDS-PAGE analysis of purified WT and mutant MtgA. Each lane contains 3 µg of protein. Right) Magnified crystals of MtgA viewed through a polarizing filter.

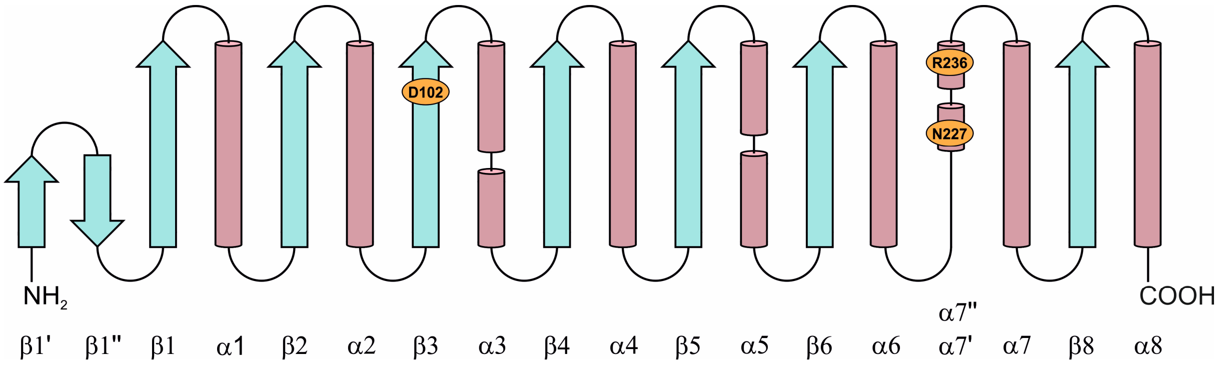

Figure S2. Secondary structure topology plot of MtgA illustrating its $(\beta/\alpha)_8$ TIM barrel fold. β-sheets are represented as blue arrows, α-helices as red cylinders. Main secondary structure elements are numbered consecutively from 1 to 8. MtgA contains two additional small β-sheets at the N-terminus. Furthermore, β-sheet 7 is replaced by a random coil and two small α-helices. A selection of catalytically important residues is indicated on the map with two of them occurring within this altered α7'-α7''-motive.

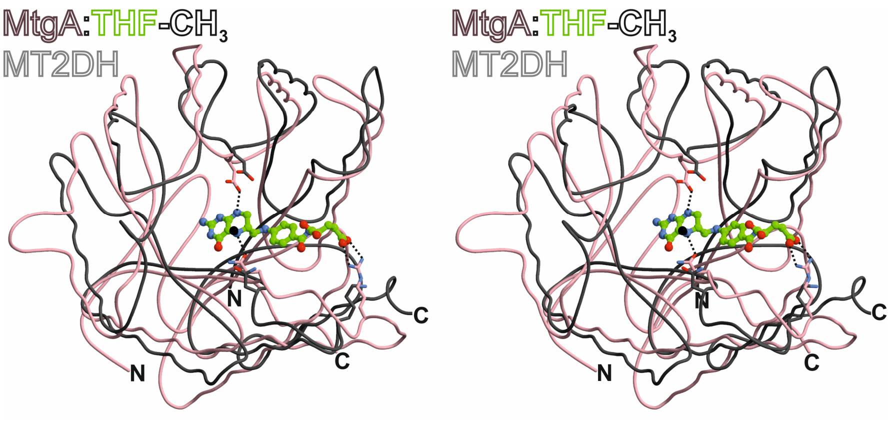

Figure S3. MtgA and the O-demethylase methyl group acceptor protein (MT2DH) from *D. hafniense* share the TIM barrel fold. Stereo view of MtgA:THF-CH3 (PDB ID: 6SJN) (pink) structurally superimposed with MT2DH (grey, PDB ID: 4O0Q).[8] Asp102 and Asn227 are conserved in MT2DH, while Arg236 is replaced by a lysine. Root mean square deviation of C_α-atoms (239 residues): 2.9 Å, sequence identity 13%, Z-score: 20.2.[10]

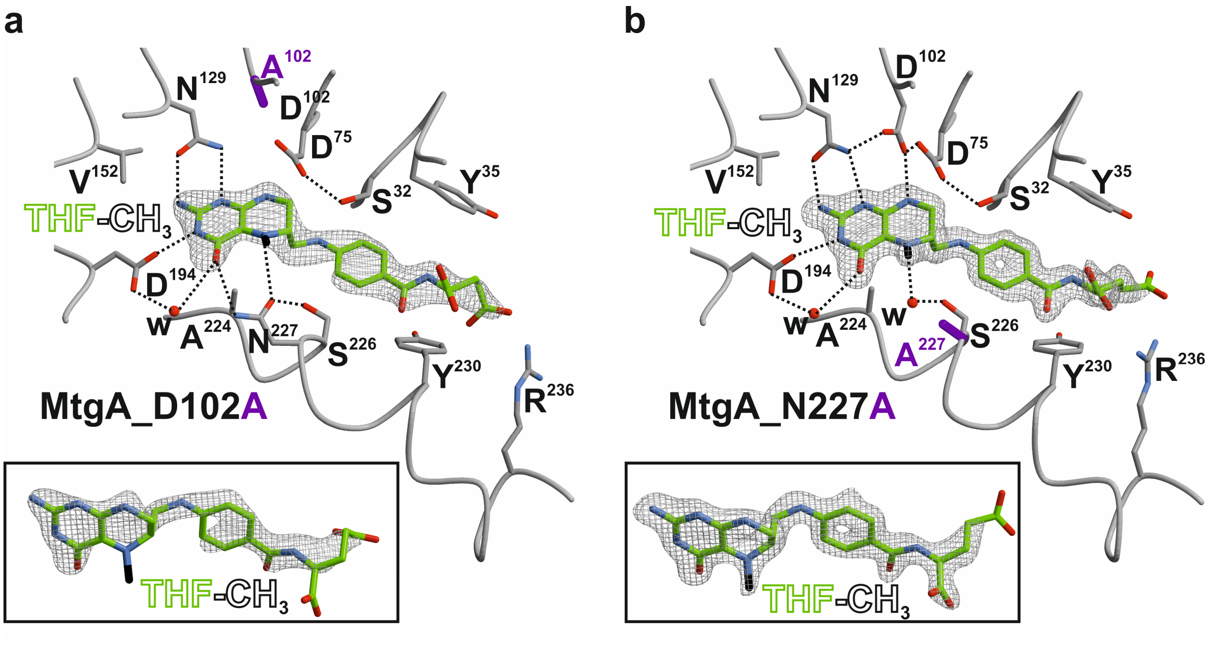

Figure S4. Active site close-up view of MtgA D102A (a) and N227A (b) in complex with THF-CH3 (PDB IDs: 6SJO and 6SJS). Residues engaged in cofactor binding are depicted as sticks and labeled by one-letter-code. The 2Fo-Fc electron density map (grey meshes, contoured to 1.0 σ) is shown for the ligand in two orientations (icon below). H bonds are drawn as black dotted lines. Color coding, contouring and labelling are according to Fig. 2.

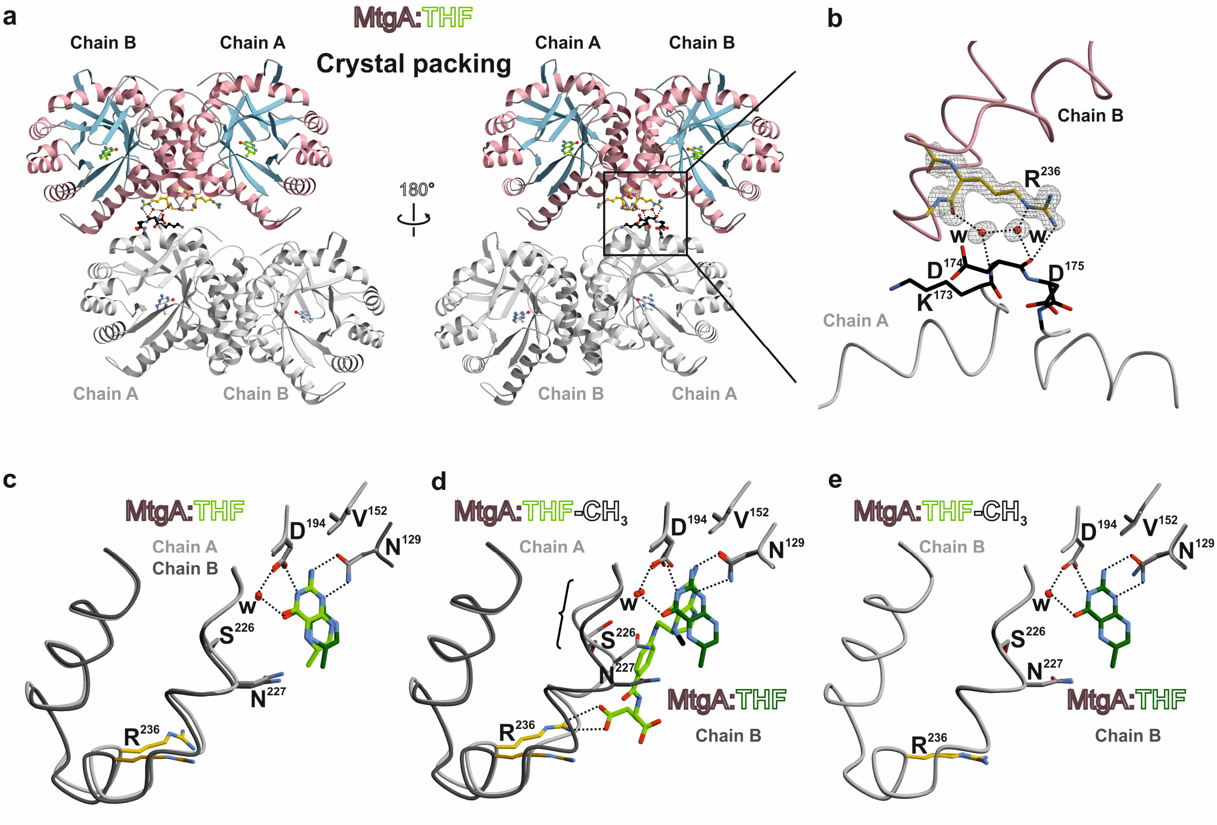

Figure S5. Crystal packing of MtgA:THF-CH_3 ($P2_12_12_1$, PDB ID: 6SJN). The structure of MtgA:THF-CH_3 was solved in two different space groups, $P2_1$ and $P2_12_12_1$. MtgA:THF-CH_3 with space group $P2_12_12_1$ depicts the cofactor only in chain A, which can be explained by crystal packing. a) The adjacent symmetry mate in MtgA:THF is shown in grey and Arg236 is depicted in yellow. Arg236 of chain B is H-bonded to main chain atoms in chain A of the neighboring dimer. A black square indicates the area for close-up views (panel b-e). b) Zoom-in of crystal contacts induced by Arg236. The $2F_o$-F_c electron density map (grey mesh) of the amino acid and two coordinating water molecules is contoured to 1.0 σ. c) Apart from Arg236, chains A and B of the MtgA:THF complex match perfectly. The guanidine side chain of Arg236 is shifted by approximately 3 Å. d) Overlay of chain A with chain B of the methyltransferase in complex with THF-CH_3 and THF, respectively. Binding of THF-CH_3 causes structural changes of the small α-helix around Asn227 (black bracket, Fig. 2e, Fig. S2). Arg236 forms the boundary of the specificity pocket and is H-bonded to the γ-glutamyl moiety of THF-CH_3. e) Crystal packing misaligns Arg236 in chain B, prohibiting proper coordination of THF-CH_3. Color coding, contouring and labelling are according to Fig. 2.

3 Supporting tables

Table S1 Primer sequences used for cloning and mutagenesis. Introduced restriction sites are highlighted in green, altered codons in red.

	Primer	Nucleotide sequence (5' → 3')
Cloning	*Dh*MtgA BamHI fw	CCA GGA TCC TTC AAG TTT ACT GCC CAA CAA C
	*Dh*MtgA PstI rev	CCA CTG CAG TTA AAA GAT TTT CAG TAA CGG ATG TTC
Mutagenesis	*Dh*MtgA D102A fw	ATC GGG AGA GAT CGA AGC CAG GAG AAA TGG AGC
	*Dh*MtgA D102A rev	GCT CCA TTT CTC CTG GCT TCG ATC TCT CCC GAT
	*Dh*MtgA N227A fw	TA CAC GGC GGC ACT CGG CGC GCA ACC GC
	*Dh*MtgA N227A rev	GC GGT TGC GCG CCG AGT GCC GCC GTG TA
	*Dh*MtgA N227D fw	CAC GGC GTC ACT CGG CGC GCA ACC
	*Dh*MtgA N227D rev	GGT TGC GCG CCG AGT GAC GCC GTG
	*Dh*MtgA R236A fw	GG GGT GCC TTT GCT GGC CAT TTT TTT CCA CAG GTA CA
	*Dh*MtgA R236A rev	TG TAC CTG TGG AAA AAA ATG GCC AGC AAA GGC ACC CC

Table S2 Sitting-drop crystallization parameters that led to diffracting crystals.

MtgA	Additive	Additive concentration [mM]	Protein-reservoir ratio [µL/µL]	Reservoir
WT	Apo	-	0.2/0.1	100 mM HEPES pH 7.5, 27 % (w/v) PEG 3350
	THF	5	0.2/0.1	100 mM Bis-Tris pH 6.5, 26 % (w/v) PEG 3350
	THF-CH_3	5	0.2/0.2	100 mM HEPES pH 7.5, 26 % (w/v) PEG 3350
WT ($P2_1$)	THF-CH_3	10	0.3/0.1	100 mM Tris pH 8.0, 29 % (w/v) PEG 3350
WT (SeMet)	Apo	-	0.2/0.2	100 mM HEPES pH 7.8, 21 % (w/v) PEG 3350
D102A	THF-CH_3	5	0.2/0.2	100 mM Bis-Tris pH 6.2, 29 % (w/v) PEG 3350
N227A	Apo	-	0.3/0.1	100 mM HEPES pH 7.5, 19 % (w/v) PEG 3350
	THF	10	0.2/0.1	100 mM HEPES pH 7.5, 24 % (w/v) PEG 3350
	THF-CH_3	10	0.2/0.1	100 mM HEPES pH 7.8, 22 % (w/v) PEG 3350

Table S3 Crystallographic data collection and refinement statistics

	MtgA [SeMet]	MtgA	MtgA:THF	MtgA:THF-CH_3	MtgA:THF-CH_3	D102A:THF-CH_3	N227A	N227A:THF	N227A:THF-CH_3
**Crystal parameters**									
Space group	$P2_12_12_1$	$P2_12_12_1$	$P2_12_12_1$	$P2_12_12_1$	$P2_1$	$P2_12_12_1$	$P2_12_12_1$	$P2_12_12_1$	$P2_12_12_1$
Cell constants	a = 76.2<br>b = 83.8<br>c = 86.7	a = 76.1<br>b = 84.1<br>c = 86.8	a = 75.8<br>b = 83.9<br>c = 86.5	a = 75.9<br>b = 83.8<br>c = 86.6	a = 83.4<br>b = 73.1<br>c = 89.8<br>β = 113.4°	a = 77.0<br>b = 84.3<br>c = 87.6	a = 76.0<br>b = 83.9<br>c = 86.6	a = 76.0<br>b = 84.0<br>c = 86.4	a = 76.2<br>b = 84.3<br>c = 86.7
Subunits / AU[a]	2	2	2	2	4	2	2	2	2
**Data collection**									
Beam line	X06SA, SLS	X06SA, SLS	X06SA, SLS	X06SA, SLS	X06SA, SLS	X06SA, SLS	X06SA, SLS	X06SA, SLS	X06SA, SLS
Wavelength (Å)	0.979	1.0	1.0	1.0	1.0	1.0	1.0	1.0	1.0
Resolution range (Å)[b]	30-1.9<br>(2.0-1.9)	30-1.85<br>(1.95-1.85)	30-1.35<br>(1.45-1.35)	30-1.75<br>(1.85-1.75)	30-1.55<br>(1.65-1.55)	30-1.95<br>(2.05-1.95)	30-1.9<br>(2.0-1.9)	30-1.75<br>(1.85-1.75)	30-1.8<br>(1.9-1.8)
No. observations	401906	195313	493740	227947	427254	214249	172347	218088	191359
No. unique reflections[c]	82696[#]	46728	120092	55629	139692	41877	43506	55277	51389
Completeness (%)[b]	97.9 (95.8)	96.9 (99.2)	99.1 (99.6)	98.7 (99.4)	97.2 (96.3)	99.2 (99.2)	98.1 (98.9)	97.9 (95.4)	98.1 (99.2)
R_{merge} (%)[b, d]	9.4 (47.1)	8.5 (57.4)	4.8 (64.5)	6.3 (51.5)	7.1 (55.7)	9.2 (57.9)	12.6 (57.0)	9.2 (56.4)	9.3 (59.5)
I/σ (I)[b]	10.8 (3.3)	9.4 (2.3)	13.8 (2.1)	13.9 (2.7)	8.1 (2.2)	11.5 (2.6)	7.5 (3.0)	11.2 (2.4)	8.6 (2.2)
**Refinement (REFMAC5)**									
Resolution range (Å)		30-1.85	30-1.35	30-1.75	30-1.55	30-1.95	30-1.9	30-1.75	30-1.8
No. refl. working set		44379	119987	52746	132691	39770	41319	52502	48810
No. refl. test set		2336	5999	2776	6984	2093	2175	2763	2569
No. non hydrogen		4898	5351	5144	9768	4818	4867	4996	4890
No. of ligand atoms		-	26	33	132	33	-	26	46
Solvent (H_2O, ions, glycerol)		212	617	425	408	106	165	268	164
R_{work} / R_{free} (%)[e]		17.3/20.4	11.9/15.7	13.7/17.3	20.9/24.3	18.5/21.7	18.9/22.4	16.9/18.9	17.9/21.9
r.m.s.d. bond (Å) / (°)[f]		0.002/1.2	0.01/1.5	0.007/1.2	0.004/1.3	0.004/1.3	0.004/1.2	0.002/1.2	0.003/1.2
Average B-factor ($Å^2$)		26.8	19.4	23.2	21.9	31.9	23.4	18.7	22.3
Ramachandran Plot (%)[g]		98.7/1.3/0	99.3/0.7/0	99.3/0.7/0	99.3/0.7/0	99.0/1.0/0	99.0/1.0/0	98.8/1.2/0	99.0/1.0/0
**PDB ID**		**6SJK**	**6SJ8**	**6SJN**	**6SK4**	**6SJO**	**6SJP**	**6SJR**	**6SJS**

[a] Asymmetric unit

[b] The values in parentheses for resolution range, completeness, R_{merge} and I/σ (I) correspond to the highest resolution shell

[c] Data reduction was carried out with XDS and from a single crystal. [#]Friedel pairs were treated as individual reflections

[d] $R_{merge}(I) = \Sigma_{hkl}\Sigma_j \mid I(hkl)_j - \langle I(hkl)\rangle \mid / \Sigma_{hkl} \Sigma_j I(hkl)_j$, where $I(hkl)_j$ is the jth measurement of the intensity of reflection hkl and $\langle I(hkl)\rangle$ is the average intensity

[e] $R = \Sigma_{hkl} \mid |F_{obs}| - |F_{calc}| \mid / \Sigma_{hkl} |F_{obs}|$, where R_{free} is calculated without a sigma cut off for a randomly chosen 5% of reflections, which were not used for structure refinement, and R_{work} is calculated for the remaining reflections

[f] Deviations from ideal bond lengths/angles

[g] Number of residues in favored region / allowed region / outlier region

4 Supporting references

[1] G. D. van Duyne, R. F. Standaert, P. A. Karplus, S. L. Schreiber, J. Clardy, *Journal of molecular biology* **1993**, *229*, 105.

[2] W. Kabsch, *Acta Crystallographica Section D: Biological Crystallography* **2010**, *66*, 125.

[3] G. M. Sheldrick, *Acta crystallographica. Section A, Foundations of crystallography* **2008**, *64*, 112.

[4] A. J. McCoy, R. W. Grosse-Kunstleve, P. D. Adams, M. D. Winn, L. C. Storoni, R. J. Read, *Journal of applied crystallography* **2007**, *40*, 658.

[5] K. COWTAN, *Joint CCP4 and ESF-EACBM Newsletter on Prorein Crystallogr* **1994**, *31*, 34.

[6] G. N. Murshudov, P. Skubák, A. A. Lebedev, N. S. Pannu, R. A. Steiner, R. A. Nicholls, M. D. Winn, F. Long, A. A. Vagin, *Acta crystallographica. Section D, Biological crystallography* **2011**, *67*, 355.

[7] P. Emsley, B. Lohkamp, W. G. Scott, K. COWTAN, *Acta Crystallographica Section D: Biological Crystallography* **2010**, *66*, 486.

[8] H. Sjuts, M. S. Dunstan, K. Fisher, D. Leys, *Acta crystallographica. Section D, Biological crystallography* **2015**, *71*, 1900.

[9] C. W. Goulding, D. Postigo, R. G. Matthews, *Biochemistry* **1997**, *36*, 8082.

[10] L. Holm, *Bioinformatics (Oxford, England)* **2019**.
